# From the Table to the Tumor: The Role of Mediterranean and Western Dietary Patterns in Shifting Microbial-Mediated Signaling to Impact Breast Cancer Risk

**DOI:** 10.3390/nu11112565

**Published:** 2019-10-24

**Authors:** Tiffany M. Newman, Mara Z. Vitolins, Katherine L. Cook

**Affiliations:** 1 Department of Cancer Biology Wake Forest University School of Medicine, Winston-Salem, NC 27157, USA; tnewman@wakehealth.edu; 2 Department of Epidemiology and Prevention, Wake Forest University School of Medicine, Winston-Salem, NC 27157, USA; mvitolin@wakehealth.edu; 3 Department of Surgery, Wake Forest University School of Medicine, Winston-Salem, NC 27157, USA

**Keywords:** breast cancer, Mediterranean diet, Western diet, microbiota, inflammation, mammary gland

## Abstract

Diet is a modifiable component of lifestyle that could influence breast cancer development. The Mediterranean dietary pattern is considered one of the healthiest of all dietary patterns. Adherence to the Mediterranean diet protects against diabetes, cardiovascular disease, and cancer. Reported consumption of a Mediterranean diet pattern was associated with lower breast cancer risk for women with all subtypes of breast cancer, and a Western diet pattern was associated with greater risk. In this review, we contrast the available epidemiological breast cancer data, comparing the impact of consuming a Mediterranean diet to the Western diet. Furthermore, we will review the preclinical data highlighting the anticancer molecular mechanism of Mediterranean diet consumption in both cancer prevention and therapeutic outcomes. Diet composition is a major constituent shaping the gut microbiome. Distinct patterns of gut microbiota composition are associated with the habitual consumption of animal fats, high-fiber diets, and vegetable-based diets. We will review the impact of Mediterranean diet on the gut microbiome and inflammation. Outside of the gut, we recently demonstrated that Mediterranean diet consumption led to distinct microbiota shifts in the mammary gland tissue, suggesting possible anticancer effects by diet on breast-specific microbiome. Taken together, these data support the anti-breast-cancer impact of Mediterranean diet consumption.

## 1. Introduction

It is predicted that there will be 15 million newly diagnosed cases of cancer and 12 million cancer patient deaths in 2020 [1]. As cancer mortality rises, researchers must examine possible risk factors contributing to an increase in prevalence. Lifestyle choices are a major target of this research, and it is estimated that 35% of all cancer deaths may be caused by dietary factors [2]. Diet-associated elevation of cancer risk has been specifically linked to 13 malignancies, and is most closely correlated with prostate, colorectal, gall bladder, pancreatic, endometrial, and breast cancer patient deaths [3]. Obesity is associated with 14% of cancer deaths in males and 20% in females, though dietary intake (i.e., red meat intake) regardless of body weight is heavily correlated with cancer risk and prognosis [4]. This correlation between dietary intake and cancer risk is well reported. However, our understanding of the mechanisms behind this remain unclear.

More than 12% of US women will be diagnosed with breast cancer during their lifetime, and in 2019 an estimated 268,600 new cases will be diagnosed [5]. Breast cancer risk is associated with health and lifestyle behaviors. Prevention efforts are critically important to reduce the number of invasive breast cancer diagnoses. It is estimated that approximately 30% to 50% of all cancer cases could be prevented by practicing healthy lifestyle habits and minimizing exposure to cancer risk factors [6]. Evidence continues to accumulate on the impact of dietary components and their effect on biological mechanisms that impact cancer susceptibility.

To lower the risk for cancer, diet-based recommendations include consuming a dietary pattern that consists largely of vegetables (including beans and legumes), fruits, and whole grains. In addition, recommendations include reducing exposure to the following dietary components: processed red meats, refined sugars, carbohydrates, fat, and excessive alcohol [7].

## 2. Dietary Patterns and Breast Cancer Risk

The Western dietary pattern (WeD) (also called the standard American diet, SAD) that is consumed by many in the United States is recognized for containing high amounts of refined starches, sugar, red and processed red meats, saturated fats, trans fats, and low amounts of fruit, vegetables, and whole grains. It was reported that more than half of the calories in the WeD come from highly processed foods. A cross-sectional study utilizing National Health and Nutrition Examination Survey 2009–2010 in 9317 participants who were at least 1 year of age and older revealed that nearly 58% of the energy intake, stated as almost three out of five of calories, was in the form of highly processed foods [8].

The WeD has been associated with elevated breast cancer risk. A study was conducted to evaluate the association between dietary patterns and risk of breast cancer in Spanish women. Researchers recruited 1017 women with incident breast cancer and 1017 matched healthy controls of similar age with no history of breast cancer. They utilized the Alternate Healthy Eating Index and the Alternate Mediterranean Diet score to assess diet adherence. The researchers reported that adherence to the WeD was associated with increased risk (OR high versus low adherence (95% (CI) 1.46 (1.06–2.01)) and that adherence to the MeD was related to lower risk (OR high versus low adherence (95% CI) 0.56 (0.40–0.79)) [9]. A cross-sectional study of 3584 women who were enrolled in a breast cancer screening program during October 2007–July 2008 was conducted to investigate the association between the WeD and MeD and mammographic density. Mammographic density was categorized as (1) less than 10%, (2) 10–25%, (3) 25–50%, and (4) greater than 50% The researchers reported that women who had higher adherence to the WeD were more likely to have high mammographic density (*n* = 242 (27%)), compared to women with low adherence to the WeD (*n* = 169 (19%)) with a fully adjusted odds ratio (ORQ4 vs. Q1) of 1.25 (95% confidence interval (CI) 1.03–1.52). It was reported that the association was specific to overweight and obese women (adjusted ORQ4 vs. Q1 (95% CI 1.41 (1.13–1.76)). There was no association between MeD and mammographic density [10]. Elevated mammographic density is associated with increased breast cancer risk.

The WeD has been reported to promote inflammation [11,12]. Extreme blood glucose variations created by consuming calorie-dense processed foods has been shown to stimulate inflammation and increase oxidative stress, as evidenced by increased circulating concentrations of pro-inflammatory molecules. Arachidonic acid derived *n*-6 eicosanoids from refined vegetable oils increase the production of pro-inflammatory cytokines.

The Mediterranean dietary (MeD) pattern has consistently been considered one of the healthiest dietary patterns because of its inclusion of an abundance of plant-based foods and the lack of processed foods [13]. The MeD emphasizes consumption of vegetables, fruits, whole grains, seeds and nuts, olive oil, lean protein (fish, poultry), and eggs, and is low in processed meat, red meat, and refined sugars. Adherence to the MeD was reported to protect against diabetes, cardiovascular disease, and some cancers [14]. Studies were conducted to evaluate the relationship between adherence to the MeD and breast cancer risk, and inverse associations have been reported [15,16,17,18,19,20]. Importantly, adherence to a MeD has been reported to decrease incidence of breast cancer of all subtypes and to decrease risk of breast cancer recurrence [15,21].

Turatui et al. (2018) investigated the association between adherence to the Mediterranean diet and breast cancer risk utilizing hospital-based case-controls, and found that adherence to the MeD was associated with a reduced breast cancer risk [22]. The researchers identified 3034 breast cancer cases and 3392 controls who were hospitalized for an acute disease. Adherence to the Mediterranean diet was measured using the Mediterranean diet score (MDS), which sums the components of the MeD. Scores range from 0 (least adherent) to 9 (highly adherent). Compared to a MDS of 0–3, the odds ratios (ORs) for breast cancer were 0.86 (95% confidence interval (CI), 0.76–0.98) for a MDS of 4–5 and 0.82 (95% CI, 0.71–0.95) for a MDS of 6–9 (*p* for trend = 0.008). When the researchers excluded the alcohol component from the scoring it did not greatly modify the ORs (e.g., OR = 0.81, 95% CI, 0.70–0.95, for MDS ≥ 6).

The MeD was reported to reduce the risk of estrogen receptor negative (ER–) breast cancer, although in the same study, a non-significant weak relation with ER positive (ER+) and total breast cancer risk was found. This was a cohort study that followed 62,573 women aged 55–69 in the Netherlands for over 20 years. Adherence to the MeD was assessed by the alternate Mediterranean Diet Score, which excluded alcohol. The analysis was conducted on 2321 incident breast cancer cases and 1665 sub-cohort participants. The researchers found an inverse association between MeD adherence and risk of ER− breast cancer, with a hazard ratio of 0.60 (95%CI, 0.39–0.93) for high MeD adherence versus low MeD adherence (*p* for trend  =  0.032). These researchers also conducted a meta-analyses of their results along with another published cohort study, and reported that hazard ratios for high versus low MeD adherence were 0.94 for total postmenopausal breast cancer, 0.98 for ER+, 0.73 for ER−, and 0.77 for ER − progesterone receptor negative breast cancer [16,23,24,25]. The largest intervention trial to test the impact of the Mediterranean diet on important outcomes was the Prevención con Dieta Mediterránea (PREDIMED). PREDIMED was a 5-year study conducted in Spain that evaluated the effects of the Mediterranean diet on primary prevention of cardiovascular disease in subjects at high risk [26]. PREDIMED was a multicenter, randomized, controlled clinical trial of 7447 participants who were randomized to one of three diets: MeD supplemented with extra-virgin olive oil (EVOO); MeD supplemented with mixed nuts; or a low-fat control diet. The results of an interim analysis prompted early stoppage of the trial after a median follow-up of 4.8 years due to reductions in the cardiovascular event rates in both intervention groups. The PREDIMED investigators also reported that consumption of the MeD supplement with EVOO and tree nuts lowered the risk of postmenopausal breast cancer by 51% [15]. Breast cancer incidence was a secondary outcome of the trial for women without a prior history of breast cancer (*n*  =  4152). After a median follow-up of 4.8 years, 35 confirmed incident cases of breast cancer were reported. Observed rates (per 1000 person-years) were 1.1 for the MeD with EVOO group, 1.8 for the MeD with nuts group, and 2.9 for the control group. The multivariable-adjusted hazard ratios compared to the control group were 0.32 (95% CI, 0.13–0.79) for the MeD with EVOO group and 0.59 (95% CI, 0.26–1.35) for the MeD with nuts group.

This is not surprising, as key constituents of the MeD are fruits and vegetables that are rich in antioxidants, and their consumption is associated with lower oxidative stress and inflammation [11]. Vegetables, fruits, lean protein, and monounsaturated fatty acids contained in olive oil were reported to reduce postprandial glucose variations and pro-inflammatory molecule secretion [27]. The *n*-3 fatty acids contained in fish, fish oil, and nuts that are also components of the MeD have anti-inflammatory effects, and thereby reduce arachidonic-acid-derived eicosanoids [28].

## 3. Diet and the Gut Microbiome

A major mechanism for dietary influence is found in the microbial balance of the gut. While the gut microbiome is impacted by many factors, including genetics, dietary intake is the primary modulator of the composition and diversity of the microbiota, and health outcomes can be impacted through this interaction [29]. Dietary intake is capable of causing shifts in gut microbial composition in both obese and lean individuals [30]. Microbial shifts are related to animal and plant-based diets, intake of fat, protein, fiber, and carbohydrates, as well as phytochemical intake [31]. In healthy individuals, the gut microbiome is characterized by having a high species richness and diversity [32]. These bacteria contribute to a healthy state by competition with pathogens, as well as assisting with the digestion of food for production of bioactive components [33]. The balance found in the gut microbiome of a healthy individual is considered anti-inflammatory. However, dysbiosis, or imbalance, in the gut microbial populations can lead to inflammation through T cell responses generated in the Peyer’s patches of the gut lamina propria [34]. Inflammation is a risk factor for cancer, a correlation that was first illustrated by Rudolf Virchow’s studies in 1863 [35]. It is now estimated that 25% of all cancer cases can be etiologically linked to inflammation and bacterial infection [36].

The implications of dietary intake and inflammation as cancer risk factors are frequently examined. However, the exact mechanisms linking these factors remain under investigation. Modulation of the gut microbiome presents a potential mechanism, which could mediate tumor-promoting inflammation in response to dietary intake.

The composition of the gut microbial population is influenced by factors including geography, maternal delivery method, ethnicity, and lifestyle [37]. Lifestyle primarily influences both long- and short-term gut microbiota composition through diet. Bacterial abundance is modulated by dietary components, such as protein, fat, and carbohydrates [38]. A high-fat diet independent of obesity results in alterations of bacterial populations at the phylum level, including a decrease in *Bacteriodetes* and increases in *Proteobacteria* and *Firmicutes* in mouse models, indicating that gut microbial balance reacts primarily to diet, and not to obesity [30,39,40]. These results were confirmed in humans, signifying that the observed shift in the *Bacteroidetes*/*Firmicutes* ratio is translational to what is shown in the human gut microbiota [41]. Differential patterns of shifted gut microbiota composition are associated with the habitual consumption of animal fats, vegetable-based diets, or diets that are high in fiber [42,43,44,45]. In humans, a study indicated that adherence to a Mediterranean diet decreased *Ruminococcus* and increased fecal *Lachnospira* and *Prevotella* in fecal samples, compared with samples from people consuming an omnivore diet [46], demonstrating that consumption of MeD shifts the gut microbiome. This was further confirmed using a non-human primate model, where monkeys consumed WeD or MeD for 31 months. The WeD and MeD were formulated to be isocaloric with respect to protein, fat, and carbohydrates, and identical in cholesterol content (~320 mg/2000 calories/day). The WeD was formulated to be similar to that consumed by American women age 40–49, as reported by the USDA [47]. Specifically, fat and protein were derived from animal sources; the diet was high in sodium and saturated fats, and the WeD was low in monounsaturated (MUFA) and *n*-3 PUFA fatty acids [47]. The MeD contained protein and fats derived from plant sources, some lean protein from fish and dairy, and high MUFAs derived from the olive oil content [48,49]. The MeD *n*-6/*n*-3 fatty acid ratio was similar to a traditional hunter-gatherer type diet [50]; that is, higher in complex carbohydrates and fiber, and lower in sodium and refined sugars. Feces from non-human primates consuming a WeD versus MeD displayed distinct gut microbiota populations, with MeD-fed monkeys having a significantly higher gut microbiota diversity than WeD-consuming subjects. Analyses into specific fecal genera modulated by dietary pattern indicated significantly higher abundance of *Lactobacillus*, *Clostridium*, *Faecalibacterium*, and *Oscillospira* genus, and lower abundance of *Ruminococcus* and *Coprococcus* genus bacteria in MeD consumers when compared to WeD-consuming female cynomolgus monkeys [51].

## 4. Gut Microbial Dysbiosis as a Driver of Inflammation

Diet-mediated shifts in the gut microbiome can induce a state of “dysbiosis”, which refers to change in the structural or functional balance of the gut microbial population. Dysbiosis is indicated in many human diseases, including obesity, auto-immunity, diabetes, allergies, neurological disorders, infection, and chronic inflammatory conditions [52,53]. Inflammatory conditions associated with dysbiosis in the gut microbiome rely on interactions between the immune cells found in the Peyer’s patches of the gut lining and the gut microbial contents.

**Figure 1:**
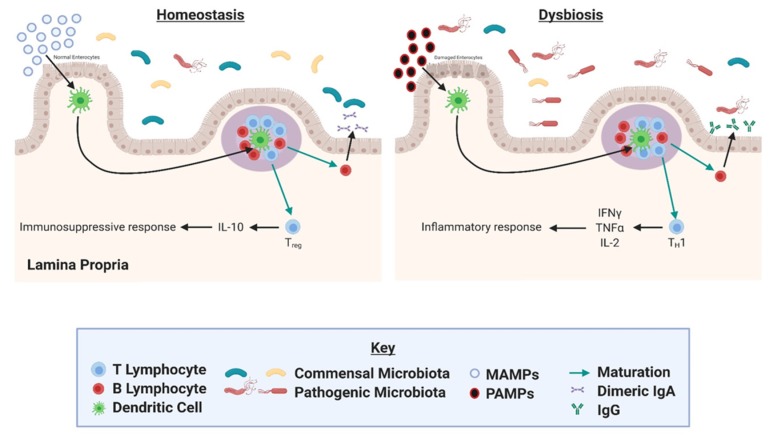
Potential microbiota signaling mechanism in the gut. Dendritic cells in the gut lining sample bacterial antigens. The dendritic cells present the antigens to T cells in the Peyer’s patches (represented by purple circle). Antigen presentation can induce T-cell differentiation, regulating inflammation. Figure was created with BioRender.com.

The gut is composed of a series of physical and functional barriers (mucus layer, epithelial layer) that serve to keep gut contents and antigens separate from the immune cells of the gut-associated lymphoid tissue (GALT) [54]. The existence of these barriers is crucial for the avoidance of inflammation in the gut lining, as the GALT constitutes the body’s largest mass of lymphoid tissue [55]. The GALT is separated from the gut microbiota by a layer of gut epithelial cells, connected to each other with tight junctions that prevent the exposure of antigens to immune cells in the GALT [56]. However, the presence of microbiota does influence the GALT (Figure 1), and this is evidenced by the lesser degree to which Peyer’s patches and Mesenteric lymph nodes are present in germ-free mice lacking gut microbial colonization [55].

Dendritic cells in the gut lining sample bacterial antigens in the gut by extending processes between tight junctions of gut epithelial cells. The dendritic cells are then able to present the antigens to T cells in the Peyer’s patches (aggregates of lymphoid tissue found in the small bowel). This presentation can induce T cell differentiation toward an immunosuppressive Treg cell phenotype in response to molecular patterns associated with commensal bacteria, or into an inflammatory phenotype in response to pathogenic particles [57]. In this way, dysbiosis of the gut microbial populations may induce an inflammatory response.

## 5. Gut Microbiome Shifts Can Correlate with Breast Cancer Risk

It is estimated that approximately 15%–20% of carcinogenesis is related to microbial infection, with *Helicobacter pylori* and human papillomavirus being widely accepted for their tumorigenic roles. However, growing evidence suggests that this estimate is low, and general microbial dysbiosis may also be accountable for tumorigenesis [33]. As previously mentioned, approximately 35% of all cancers are associated with dietary intake. This statistic includes 75% of prostate, 70% of colorectal, 50% of endometrial, 50% of breast, 50% of pancreatic, and 50% of gall bladder carcinomas, among others [3]. In each of these diet-associated cancers, microbial-mediated mechanisms are possible modulators of carcinogenesis and tumor aggressiveness.

Breast cancer pathogenesis is associated with major gut microbial shifts, and patients were shown to express enrichment in *Clostridiaceae*, *Faecalibacterium*, and *Ruminococcaceae*, and reduction in *Dorea* and *Lachnospiraceae* compared to controls [58]. The existence of gut microbial profiles associated with specific cancers suggests roles for these bacterial taxa in carcinogenesis, prognosis factors, and preventative activities, thus indicating a potential mechanism by which diet may mediate cancer risk and prognosis.

## 6. Mammary Gland Microbiome and Breast Milk

The presence of microbes in the mammary gland was first examined in the milk of lactating mothers [59]. Human breast milk is enriched in *Streptococcus*, *Staphylococcus*, *Bifidobacterium*, *Propionibacterium,* and *Lactobacillus* bacteria and assists in early colonization of the infant gut microbiome [59,60]. However, a mammary-gland-specific microbiome was identified in tissue from non-lactating women as well [61]. The origin of this mammary microbiome remains under investigation. Current theories indicate the possibility of translocation from the gut microbiome, as well as transfer from the skin through nipple–areolar orifices [62].

A healthy mammary gland microbial population has been associated with enrichment of the *Sphingomonas yanoikuyae* bacterial species. Notably, *S. yanoikuyae* may contribute to an immunomodulatory response in the breast microenvironment through expression of glycosphingolipid ligands, which activate invariant natural killer T (iNKT) cells [63]. Further study is needed to examine the exact mechanisms, beneficial properties, and potential probiotic applications of *S. yanoikuyae* in the mammary gland tissue.

In order for probiotic bacteria to instigate a beneficial response in the mammary gland, microbial response elements must be present on host tissue. Xuan et al. reported enriched expression of microbial sensors (TLR2, TLR5, TLR9, NOD1, and NOD2), downstream signaling molecules (CARD6, CARD9, and TRAF6), and antimicrobial response effectors (BPI, IL-12A, MPO, and PRTN3) in healthy breast tissue. Further investigation of these genes may provide evidence for the mechanisms by which the healthy mammary gland microbiome utilizes the host immune system to interact with host tissue [63].

## 7. Mammary Gland Microbiome and Breast Cancer

Breast cancer has been associated with dramatic changes in the mammary microenvironment and microbiome. While normal mammary tissue exhibits enrichment of microbial response element genes, Xuan et al. noted significant reduction in microbial response element expression of breast tumor tissue. This observation was paired with a reduced population of potentially probiotic *S. yanoikuyae* [63].

Further examination of the cancer-associated mammary microbiome utilized tumor samples and healthy control tissue from breast reduction surgeries. Mammary gland samples obtained from women undergoing lumpectomies, mastectomies, or breast reduction surgeries living in Canada or Ireland showed distinct genus taxa differences. See Table 1 for mammary gland microbiota populations, adapted from Urbaniak et. al. 2014. Whether the observed taxa differences or abundance differences were due to differences in body mass index or obesity is unknown, since those data were unreported in the study. Literature indicates that mammary gland microbiota differ between non-cancerous women and women with breast tumors. Women with breast cancer had elevated *Staphylococcus* in their tumor-adjacent mammary gland tissue compared to women without cancer [64]. In another study investigating the microbiome of mammary gland tissue obtained from patients with benign tumors or malignant breast cancers that controlled for patient BMI, breast tissue from those with malignant breast cancer had decreased *Lactobacillus* [62], suggesting mammary tissue dysbiosis as a possible driver of breast cancer.

## 8. Mediterranean and Western Diet Impact on Mammary Gland Microbiome

Diet is a main determinant of gut microbial diversity; however, the impact of diet on microbiota in organ sites other than the gut are underexplored. In a non-human primate model, our group demonstrated that consumption of a WeD or MeD modulated mammary gland microbiota and metabolite profiles. Consumption of MeD resulted in an approximate 10-fold increase in mammary gland *Lactobacillus* abundance when compared with mammary tissue from WeD-fed monkeys [65]. Moreover, mammary glands from MeD-fed monkeys had higher levels of select bile acid metabolites and increased bacterial-processed bioactive compounds when compared with plasma level and WeD-fed monkey mammary tissue. These data suggest that diet directly influences microbiome populations outside of the intestinal tract in distal sites such as the mammary gland. Our study demonstrates diet impacts the mammary gland microbiome, establishing an alternative mechanistic pathway for breast cancer prevention [65].

Literature indicates that treatment of MCF-7-estrogen-receptor-positive breast cancer cell line with either chenodeoxycholate (CDCA) or glycochenodeoxycholate (GCDCA) resulted in opposed effects on proliferation; GCDCA increased MCF7 proliferation, while treatment with CDCA reduced proliferation [66]. We specifically observed distinct changes in bile acid metabolites in mammary tissue of MeD-fed monkeys. We observed increased CDCA metabolite levels (previously shown to be anti-proliferative) in mammary glands of monkeys fed a MeD, with no significant differences in GCDCA (previously shown to stimulate breast cancer cell proliferative), possibly implicating dietary regulation of bile acid metabolites as a breast cancer prevention mechanism. We showed enrichment of taurocholate (TCA) and glycocholate (GCA) metabolites in mammary glands from MeD-consuming monkeys when compared with mammary gland tissue from Western diet consuming monkeys. These conjugated bile acids were previously demonstrated to be modified by the presence of microbes [67,68,69]. Concurrent analysis of circulating plasma bile acid metabolites in matched monkeys showed no significant regulation of GCA, TCA, or CDCA by diet, suggesting a possible mammary-gland-specific microbial regulation of bile acid metabolites. *Lactobacillus* contains bile salt hydrolase activity that modifies secondary bile acid metabolite generation. These secondary bile acid metabolites can then serve as agonists for farnesoid X receptors [70]. The enrichment of *Lactobacillus* in the mammary glands of MeD-fed monkeys may then increase mammary-gland-specific bile acid metabolite-mediated activation of farnesoid X receptor signaling for potential anticancer properties. In the clinical setting, postmenopausal women with newly diagnosed breast cancer displayed increased plasma deoxycholate (DCA) when compared with control plasma samples taken from healthy, age-matched, and BMI-matched women [71,72]. Previous studies demonstrate that DCA is a mutagenic compound [73]. No significant changes in DCA bile acid metabolite concentrations were observed in the mammary glands; however, there was a trend for decreased DCA in the plasma of MeD-fed monkeys [65].

**Figure 2:**
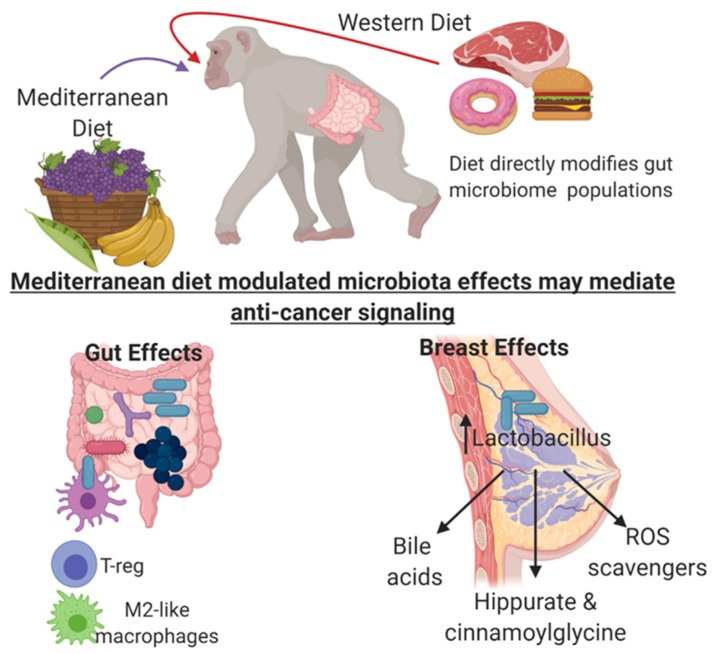
Potential microbial-modified signaling regulated by Mediterranean Diet (MeD) consumption that may regulate breast cancer risk. Immune cell sampling of probiotic *Lactobacillus* species induced by MeD consumption may promote systemic anti-inflammatory signaling. Dietary metabolites directly influence mammary gland *Lactobacillus* abundance, modify bile acid metabolites, increase bacterial-processed bioactive compounds, and increase antioxidant compounds. Figure was created with BioRender.com.

Literature demonstrates a clear association between MeD-derived phenolic and phytochemical components and important health benefits [74]. Our studies demonstrated that monkeys consuming a MeD had significantly elevated levels of mammary gland conjugated phenolic compounds, including p-cresol sulfate (tyrosine metabolite), hippurate, cinnamoylglycine, and 3-hydroxy-3-phenylpropionate (phenylalanine metabolites), and indole-containing substances (tryptophan metabolites), such as indolin-2-one, 3-indole sulfate, and indolepropionate, that was not observed in the mammary gland of the WeD-fed group [65]. While it is possible that these regulated polyphenol metabolites could have come from MeD components (olive oil, fruits, and vegetables enriched in the MeD), there were no significant differences in tryptophan, phenylalanine, or tyrosine metabolite levels in the mammary glands (parental amino acid metabolite). This then suggests that these metabolite changes may reflect changes in microbial metabolism localized within the mammary glands influenced by the two diet regimens. Further supporting the role of microbial-mediated bioactive compound generation, there were no changes in plasma metabolite levels of hippurate, cinnamoylglycine, 3-hydroxy-3-phenylpropionate, or phenylalanine by dietary pattern, suggesting the microbial modulation of these metabolites may be localized to the mammary gland tissue. We did observe significant differences in the circulating plasma metabolite concentrations of tyrosine and tryptophan-based microbial-regulated phenolic compounds (but not phenylalanine-based compounds), indicating gut-microbiome-dependent systematic effects on these two families of microbial compounds [65]. Studies comparing and contrasting the plasma metabolite profiles between conventional and germ-free mice demonstrated the critical reliance on bacterial presence for the formation of hippurate, p-cresol sulfate, cinnamoylglycine, and 3-hydroxyl-3-phenylpropionate [75]. Taken together, these data support the role of bacteria in generating metabolites that may affect localized signaling; however, for the most part, the molecular mechanism of these microbial-mediated bioactive compounds remains unknown, especially in breast tissue, and needs to be explored in future studies. An overview of the potential microbial-modified signaling regulated by diet is shown in Figure 2.

## 9. The Microbiome as an Emerging Target for Cancer Therapy

As evidence for diet-mediated microbial mechanisms in tumorigenesis grows, targeting the gut microbial contents for cancer treatment has garnered interest. Prebiotic and probiotic supplements are common selections for this goal. Prebiotics consist of indigestible food ingredients, often fibers, which are used to specifically stimulate the growth or activity of commensal gut microbiota to benefit gut health [33]. Inulin is an example of a prebiotic treatment, which is used to stimulate the growth of *Bifidobacterium* in the gut [76]. *Bifidobacterium* is commonly administered in probiotics and has been associated with an anti-inflammatory response in the gut. These supplements encourage a shift toward homeostasis in the gut microbial environment, in which the maintained balance prevents accumulation of pathogenic bacterial species and encourages an immunosuppressive reaction in the GALT [76].

**Figure 3:**
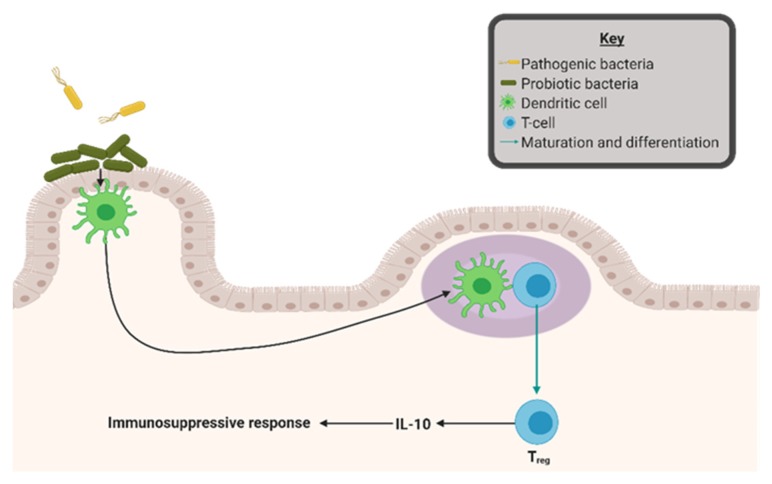
Probiotic interactions in the gut can modify inflammation. Probiotic bacterial species adheres to the gut lining, strengthening the gut epithelial barrier, and allows for competitive inhibition of pathogenic microorganisms. Additionally, probiotic bacteria produce anti-microbial substances promoting anti-inflammatory response in the gut-associated lymphoid tissue. Figure was created with BioRender.com.

Probiotics directly treat gut dysbiosis by supplementation with live commensal bacterial species, often consisting of *Lactobacillus* and *Bifidobacterium*. While the possibility of direct correlation between probiotics and cancer reduction remains under investigation, several cancer risk factors have been successfully abridged by probiotic supplementation. Probiotics are linked to altered glycemic control, contributing to decreased fasting blood glucose and insulin levels [77]. Additionally, strong correlation exists between probiotic treatment and reduced local and systemic inflammation. Ulcerative colitis patients receiving *Lactobacillus*-based probiotics exhibited a reduction of circulating pro-inflammatory cytokines and successfully maintained remission status [78,79]. *Bifidobacterium infantis*, another common probiotic species, was attributed with reduction in both gut mucosal and systemic inflammation [80]. As shown in Figure 3, the proposed mechanism for these activities relies on adherence of probiotic bacterial species to the gut lining. This adherence strengthens the gut epithelial barrier and allows for competitive inhibition of pathogenic microorganisms. Additionally, probiotic bacteria were shown to produce anti-microbial substances, which encourage an anti-inflammatory response in the gut-associated lymphoid tissue [81].

The use of probiotics in the treatment of cancer remains a relatively new idea. However, evidence for the potential success of this method is accumulating. The use of *Lactobacillus* probiotics has been successful in mouse syngeneic 4T1 triple negative breast cancer models. Oral supplementation with *Lactobacillus helveticus*-fermented milk in this model has resulted in increased cancer cell apoptosis and reduced production of pro-inflammatory cytokines, while treatment with *Lactobacillus acidophilus* has been associated with tumor growth retardation and reduced systemic inflammation [82,83]. Milk fermented with *Lactobacillus casei* reduced 4T1 tumor growth, vascularization, invasion, and metastasis [84,85]. These data suggest a role for probiotics in the reduction of tumor size and aggressiveness in a mouse model of triple negative breast cancer.

A third emerging option for targeting the gut microbiome is dietary intervention. In this category, polyphenols, such as those found in grapes, green tea, and pomegranate, are an area of interest. Dietary polyphenols act as antioxidants, and sometimes even as antibiotics. While their structure makes bioavailability through gut absorption limited, microbial hydrolysis of glycosides assists this process [31]. Treatment with green tea polyphenolic compounds has successfully reduced gut microbial presence of pathogenic *Clostridium perfringens*, *Clostridium difficile*, and *Bacteroides* in animal models [86]. Ellagic acid, a polyphenolic compound found in berries and nuts, is broken down by the gut bacteria and appears to have anticancer effects [33]. Further studies are needed to determine the exact changes in the gut microbial composition that occur in response to ellagic acid and other dietary polyphenols. We and others have also demonstrated that MeD diet consumption elevated gut [51] and mammary gland [65] *Lactobacillus* populations, possibly supporting a gut-mammary gland signaling axis or suggesting similar dietary regulation of *Lactobacillus* in both tissue types. The metabolomics analysis of paired breast tissue indicated MeD-consumption-modified mammary gland bile acid metabolites, microbial-processed bioactive compounds, and antioxidant metabolites that were observed only in the breast tissue and not seen regulated in circulating plasma, supporting the concept of a localized mammary microbiota niche [65]. Taken together, these data highlight the pleiotropic signaling in the breast tissue mediated by MeD consumption and demonstrate the need for further research to be performed to determine the origin and regulation of the breast tissue microbiome.

## 10. Discussion and Future Directions

Epidemiological data supports the critical impact of dietary pattern on breast cancer risk; WeD consumption elevates breast cancer risk, while consumption of a MeD reduces breast cancer risk. These could be due to several possible key molecular mechanisms; however, we propose that the regulation of the gut and mammary microbiome may be a key influencer on the anticancer role of MeD. Current research detailing population shifts of the gut microbiome suggests a potential mechanism for dietary influence on cancer risk through inflammation. Understanding this mechanism allows for utilization of the gut microbial composition as a target for cancer prevention and treatment. In this way, restoration of balance to the gut microbiome may prove a viable option for reduction of diet-mediated cancer risks and prognosis factors.

As the discussion of the gut microbiome in cancer progresses, the focus will likely shift to include the diet-mediated roles of other tissue microbiomes in tumorigenesis. This is of particular interest in breast cancer, as labeled oral probiotics were identified in the breast milk, suggesting that gut bacteria may be able to travel to the mammary gland [87]. Our group has previously identified distinct changes in mammary gland microbial composition in response to diet [65], which may be due to a similar mechanism. Developments such as these will be crucial as we work to determine the exact gut microbial mechanisms at play, allowing us to expound upon the pathways by which food consumed at the table can influence a tumor.

## Figures and Tables

**Table 1:** Microbiota genus proportional abundance found in human mammary gland samples (adapted from Urbaniak et al. 2014 [64]).

Microbiota Genus	Canadian Breast Tissue (% of Microbiota Population)	Irish Breast Tissue (% of Microbiota Population)
*Bacillus*	11.4%	<2%
*Acinetobacter*	10%	<2%
*Enterobacteriaceae*	8.3%	30.8%
*Pseudomonas*	6.5%	5.3%
*Staphylococcus*	6.5%	12.7%
*Propionibacterium*	5.8%	10.1%
*Prevotella*	5%	<2%
*Listeria*	<2%	12.1%
